# Electrical transport and dielectric relaxation mechanism in Zn_0.5_Cd_0.5_Fe_2_O_4_ spinel ferrite: A temperature- and frequency-dependent complex impedance study

**DOI:** 10.1016/j.heliyon.2024.e34155

**Published:** 2024-07-06

**Authors:** Raheel Mumtaz, Waqar Hussain Shah, Yousaf Iqbal, Hayat Ullah, Ghulam Asghar, Mubushar Hussain, Mostafa R. Abukhadra, Ahmed M. El-Sherbeeny

**Affiliations:** a Department of Physics, University of Poonch Rawalakot, 12350, Azad Kashmir, Pakistan; b Department of Physics, Women University of Azad Jammu and Kashmir Bagh, Pakistan; c Physics Division, Pakistan Institute of Nuclear Science and Technology, P.O. Nilore, Islamabad, 45650, Pakistan; d Materials Technologies and Their Applications Laboratory, Faculty of Science, Beni-Suef University, Beni Suef City, Egypt; e Industrial Engineering Department, College of Engineering, King Saud University, P.O. Box 800, Riyadh, 11421, Saudi Arabia

**Keywords:** Dielectric relaxation, Polaron hopping, Complex impedance, Spinel, Nyquist plots

## Abstract

In the present study, the frequency-dependent dielectric relaxation and electrical conduction mechanisms in sol-gel-derived Zn_0.5_Cd_0.5_Fe_2_O_4_ (ZCFO) spinel ferrite were studied in the temperature range of 343–438 K. The formation of the ZCFO spinel ferrite phase with space group Fd3m was confirmed by X-ray diffraction analysis. The dielectric relaxation and electrical conduction mechanisms were studied using complex impedance spectroscopy (CIS). In the Nyquist plots, depressed semicircles were fitted with an equivalent circuit model with configuration (R_GB_Q_GB_) (R_G_Q_G_), signifying the contributions from grain boundaries and grains to the charge transport mechanism in the sample. The frequency-dependent AC conductivity was found to follow Jonscher's power law, and the frequency exponent term depicted the overlapping large polaron hopping (OLPH) model as the dominant transport mechanism. The activation energies for conductivity, electric modulus and impedance were calculated to identify the nature of the charge carriers governing the relaxation and conduction mechanisms in the prepared sample. Complex modulus studies confirmed the non-Debye type of dielectric relaxation, whereas tangent loss and dielectric constant analyses confirmed the thermally activated hopping mechanism of charge carriers in Zn_0.5_Cd_0.5_Fe_2_O_4_ spinel ferrite.

## 1 Introduction

Spinel ferrites with the general formula AFe_2_O_4_ (where A = Mn, Zn, Ni, Cu, Co, Mg, etc.) have evolved into an important class of magnetic materials because of their rich electrical and magnetic properties, which depend upon several parameters, such as the preparation method, microstructural changes, and differences in ionic radii and chemical compositions [1,2]. Spinel ferrites are sometimes referred to as ferro-spinels because the crystal structure of these ferrites is quite similar to that of the mineral spinel MgO.Al_2_O_3_. It has 56 ions per unit cell. Oxygen ions with a radius of 1.3 Å are quite closely packed in the FCC arrangement, and very small metal ions with a radius of 0.7–0.8 Å occupy the free space between them. There are two types of spaces. One site is referred to as the tetrahedral (A) site because it is located at the center of the tetrahedron, which has oxygen ions at its corner, and the other site is referred to as the octahedral (B) site because the corners of the octahedron are occupied by surrounding oxygen ions [3]. Spinel ferrites are metal oxides with twice as many A sites as cationic B sites in a face-centered cubic (FCC) structure in which oxide ions are very closely packed with various degrees of freedom accessible to enhance the chemical properties of the material. Based on the favourable occupancy of metal ions at these sites, spinel ferrites are classified as normal spinel, mixed spinel or inverse spinel structures [4]. If the A site is occupied by a divalent cation, then it will form a normal spinel structure, and if the divalent cation only occupies the B site, it will form an inverse spinel structure [[5], [6], [7], [8]]. The magnetic, structural and electrical parameters of these types of spinel ferrites can be tailored by doping with different magnetic divalent or trivalent metallic ions at either the B site or the A site to create alloy ferrites. The mixture formed by this process may cause a reduction in size to make fine nanoparticles with different physicochemical characteristics [5]. The cationic redistribution on octahedral and tetrahedral sites can result in new electrical and magnetic properties. Recently, cationic redistribution has been reported to induce spin-glass and cluster-glass states in Cu-doped ZnFe_2_O_4_ spinel ferrite [9].

Zinc ferrite and cadmium ferrite both exhibit normal spinel structures according to the $Fd\bar{3}m$ space group [7]. In the normal spinel ferrite structure, site B is completely filled with 16Fe^3+,^ and site A is occupied by 8 M^2+^ (divalent metallic) ions. Normal spinel has a zero inversion parameter. When B sites are filled with trivalent cations and A sites are occupied by divalent cations, this type of dissemination or arrangement is referred to as normal spinel [4]. Although both elements have interesting optical, magnetic, and electrical properties, they have received less scientific attention for unknown reasons [10]. Both zinc and cadmium ferrites exhibit antiferromagnetic behavior when they exist in bulk form, but when they are ground in fine powder form, they show ferrimagnetic properties and magnetically saturated systems with different magnetic characteristics because of negative exchange integrals and inverted spinel formation. Cd- and Zn-based ferrites are very appropriate materials for manufacturing antenna rods, loading coils, and transformer cores used in the production of electronic and microwave devices because they exhibit interesting properties, namely, high resistivity, low coercivity, fairly high mechanical hardness and negligibly small eddy current loss [11,12]. To synthesize spinel ferrites, various scientific approaches have been adopted, such as hydrothermal methods, coprecipitation, solid-state reaction approaches and many other methods, which may have some advantages while considering the different physical and chemical properties of the sample [6]. Zinc-doped cadmium ferrites were synthesized by a sol gel autocombustion approach using ethylene glycol and citric acid as complexing reagents. The benefit of ethylene glycol and citric acid as complexing agents in the approach adopted to synthesize the required material includes proper mixing of different cations at the basic atomic level to prepare pure, homogeneous, highly ordered and single-phase mesoporous ternary ferrite nanoparticles with interesting physical and chemical arrangements at comparatively lower temperatures than those suggested in different studies [[13], [14], [15], [16]].

Complex impedance spectroscopy (CIS) is a versatile approach for studying the dielectric and electrical properties of materials of different kinds [17]. The impedance data provide information about the different electrical parameters of the material. This selection is made on the basis of (i) the impedance existing in the sample, such as the inductor, resistor or capacitor connecting in parallel or series; (ii) the graphical data analysed to verify its compatibility by using a suggested circuit; and (iii) the extracted capacitance and resistance data used to verify that either they are temperature dependent or realistic when a series circuit is used. It is used not only for measuring the amplitude of capacitive and resistive responses but also for interpreting their behavior over a wide frequency range [18]. CIS has proven to be a useful tool for examining corrosion [19], fuel cells and rechargeable batteries [[20], [21], [22], [23]]. It may also be used for investigating DNA, biosensors and sensors [[24], [25], [26], [27], [28]].

Spinel ferrites based on Zn and Cd exhibit fine mesoporous forms and show high-quality catalytic and sensor properties [5]. These spinel ferrites exhibit high coercivity, significant chemical stability, high electrical resistivity and high mechanical hardness, which makes them useful for use in memory devices working on the principle of resistive switching functions, electronics for transformers, telecommunication, magnetic drug delivery carriers, microwave devices, magneto-caloric refrigeration, magnetic storage devices, power supplies, pseudocapacitive energy storage and sensors [[29], [30], [31], [32]]. Recently, Yumi et al. reported the suitability of spinel ferrite CoFe_2_O_4_ for use as an electromagnetic generator for vibration energy harvesting [33]. Moreover, the substitution of Zn in spinel ferrites has been reported to have significant effects on the electrical, dielectric and magnetic properties of the material. For instance, Nandanwar et al. investigated the structural and magnetic properties of Zn-substituted cadmium ferrite and reported a decrease in the coercivity and saturation magnetization due to Zn^2+^ substitution [34]. Zinc doping in cobalt ferrite has also been reported to decrease H_c_ due to the incorporation of nonmagnetic zinc dopants [35]. Thus, the substitution of zinc results in the conversion of hard magnetic material to soft magnetic material by reducing the H_c_ field [36]. Electrical transport properties play a crucial role in determining these applications. Charge transport studies have revealed numerous modern-day applications, such as the fabrication of wireless multiferroic memristors coupled with gaint impedance for synapse applications [37]. Moreover, spinel ferrites containing Zn^2+^ support electron hopping conduction owing to the creation of Fe^2+^ ions as a result of Zn^2+^ volatilization at high temperatures [38,39]. Thus, charge transport due to hopping over the $Fe^{2+}-O-Fe^{3+}$ network in electro-ceramic micro-constituents (grains and grain boundaries) of Zn_0.5_Cd_0.5_Fe_2_O_4_ (ZCFO) in response to an applied alternating voltage is important for determining various physiochemical characteristics for the abovementioned applications. Recently, numerous studies have been carried out on impedance spectroscopy to explore DC/AC conductivities, dielectric properties, and their correlation with the microstructure of spinel ferrites in a varying range of frequencies and temperatures, but there are few reports where the electrical, magnetic and optical properties of ZCFO have been explored [25,40]. Several reports on the structural, dielectric and electrical properties of ZCFO have been published in the literature. For instance, Somenath et al. [8] studied the conductivity of Zn–Cd ferrites. In another report, the Mossbauer and positron annihilation characteristics of Zn–Cd ferrites were explored [41], whereas the effects of composition on quadrupole splitting in ZCFO were reported by Siddique et al. [42]. Moreover, Harish et al. reported the enhanced optical and photocatalytic activity of ZCFO under sunlight [43]. Another study conducted by Valan et al. explored the effect of Cd doping on the magnetic properties of ZnFe2O4 and reported superparamagnetic and ferromagnetic behavior for lower and higher concentrations of Cd, respectively [44]. However, to the best of the authors’ knowledge, hardly any work on the dielectric relaxation and electrical transport properties of ZCFO has been reported. Therefore, it is of significant importance to study the temperature- and frequency-dependent electrical conduction mechanism and dielectric relaxation process in ZCFO spinel ferrite for different applications.

Our present study explored in detail the frequency- and temperature-dependent electrical conductivities, which include both DC/AC conductivity and impedance analysis, as well as the corresponding hopping mechanism of charge carriers explaining the charge transport process and dielectric relaxation phenomena in Zn_0.5_Cd_0.5_Fe_2_O_4_ spinel ferrite by CIS.

## 2 Materials and methods

The sol-gel autocombustion method is widely used for the preparation of magnetic nanoparticles. Zinc nitrate hexahydrate (Zn(NO_3_)_2_.6H_2_O, 99 %), cadmium nitrate tetrahydrate (Cd(NO_3_)_2_.4H_2_O, 99 %) and iron nitrate nonahydrate (Fe(NO_3_)_3_.9H_2_O, 99 %) were used as precursors for the synthesis of ZCFO. Ethylene glycol (98 %) worked as a gelation reagent, which helped in the combustion process, caused heat evolution and decreased the ignition temperature. All chemicals were purchased from Sigma Aldrich and were used without further purification. Metal nitrates, ethylene glycol and citric acid (weighed as metal ions, ethylene glycol and citric acid in a mole ratio of 1:1:2) were dissolved in distilled water according to their stoichiometric ratios. The reaction mixture was then magnetically stirred on a hot plate at 100 °C until the polymeric gel was obtained. When the gel was molded, the temperature was progressively increased for the combustion process to occur, which eventually turned the gel into powder. The resulting powder was crushed using an agate mortar and pestle and then annealed at 950 °C in an electric furnace for 6 h. ZCFO powder was pressed to form a pellet (12.4 × 1.3 mm^2^) with the help of a uniaxial hydraulic press by applying a cold pressure of 250 MPa for approximately 10 min. The prepared pellet was subjected to heat treatment at 950 °C for 4 h in an electric furnace. During each heat treatment, the heating rate was maintained at 5 °C/min while cooling was performed due to the inertia of the furnace. Silver paste was applied on the flat surfaces of the pellets, which served as contacts for dielectric and CIS measurements.

An X-ray diffractometer (XRD, Rigaku D/Max-1200X, Tokyo, Japan) was used for the acquisition of X-ray diffraction data. CuKα X-rays with a wavelength of 1.54157 Å were used to obtain the data in the angular range of 20°–80° with a step size of 0.02° per 5 s. A surface morphological investigation of the prepared ZCFO spinel ferrite was carried out using a scanning electron microscope (Maia3 TESCAN). For dielectric, conductivity, modulus and impedance analyses, the impedance data were collected by an Alpha-N impedance analyser (Novocontrol Germany) with a 500 mV sinusoidal input AC signal. The prepared sample was scanned at frequencies ranging from 50 Hz to 10 × 10^6^ Hz at various temperatures ranging from 343 K to 438 K.

## 3 Results and discussion

### 3.1 Structural analysis

**Fig. 1:**
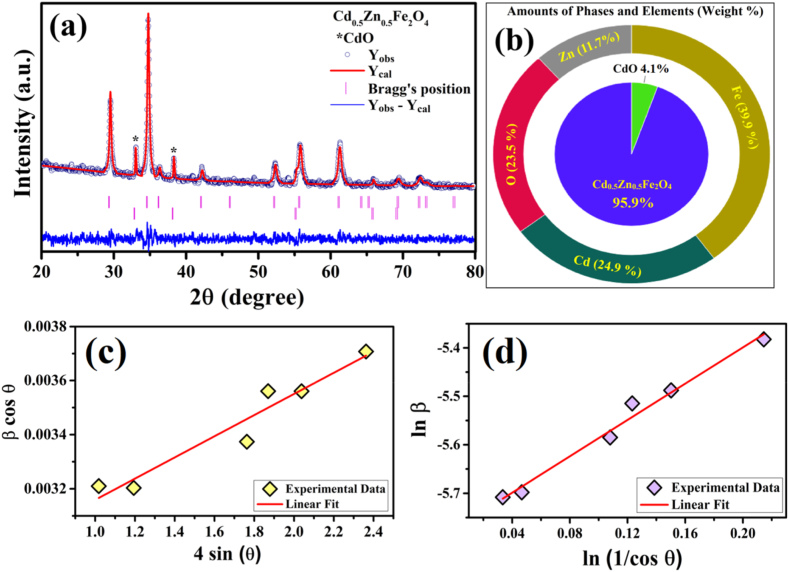
(a) Rietveld refined XRD pattern, (b) amounts of phases and elements in weight percent determined from Rietveld refinement, (c) W–H plot and (d) modified Scherrer plot of ZCFO spinel ferrite powder sintered at 950 °C.

The X-ray diffraction (XRD) patterns of the zinc cadmium ferrite (ZCFO) nanoparticles are shown in Fig. 1. The XRD pattern revealed the formation of two phases, Zn_0.5_Cd_0.5_Fe_2_O_4_ and cadmium oxide (CdO). Most of the peaks in the diffraction pattern refer to cubic Zn_0.5_Cd_0.5_Fe_2_O_4_ (JCPDS card 96-153-9596) with $Fd\bar{3}m$ space groups, whereas the peak centered at approximately 39.481° corresponds to cubic CdO (JCPDS: 96-101-1004). The presence of the CdO phase can be ascribed to the separation of reactants because of the presence of a CdO layer on the surface of salts when the temperature applied to the sample is kept below 1000 °C [45]. To determine the structural characteristics of the synthesized powder sample, Rietveld refinement analysis was performed using FullProf software on the experimentally acquired XRD data. The width of the peaks was modelled by employing FWHM (U, V, W) parameters, while the peak shape was refined using the pseudo-Voigt peak function. The background of the observed XRD data was modelled using a 12-coefficient Fourier cosine series. Various structural parameters, such as fractional atomic positions (x, y, z), lattice parameters (a, b, c, α,β,γ), isotropic displacement parameters, profile shape parameters, scale factors and asymmetry parameters, were adjusted to obtain the acceptable limits of reliability factors (R-factors) and goodness of fit ($\chi^{2}$). The results of the refinement process are presented in Table 1. The XRD pattern for ZCFO is depicted in Fig. 1(a), where the symbols represent the observed data, the red solid line is used for the calculated data, the difference between the observed and calculated data is represented by the blue solid line, and the magenta vertical bars represent the positions of the standard (hkl) peaks. The refinement results revealed the presence of Cd_0.5_Zn_0.5_Fe_2_O_4_ (95.9 %) and CdO (4.1 %) phases. The phase composition and weight percentages of the constituent elements calculated from Rietveld refinement analysis are depicted in Fig. 1(b).

**Table 1:** Experimental conditions, structural parameters and Rietveld refinement results for the Cd_0.5_Zn_0.5_Fe_2_O_4_ phase.

Crystallographic data		Data collection	
Chemical formula:	Cd_0.5_Zn_0.5_Fe_2_O_4_	Acquisition Temperature (^o^C):	25
Crystal system:	Cubic	Cu $K_{\alpha }$ wavelength (Å):	1.541874
Number of Space group:	227	Angular range (^o^):	20≤2θ≤80
Hermann-Mauguin Symbol:	Fd $\bar{3}$ m:1	Step (2θ):	0.02
a(Å):	8.5611	Rietveld data:	
b(Å):	8.5611	Software program:	FullProf Suite (Version January 2021)
c(Å):	8.5611	Profile function:	Pseudo Voigt
α (^o^):	90	Number of fitted parameters:	12
β (^o^):	90	Caglioti parameters:	U = 0.218582; V = −0.00068; W = 0.111711
γ (^o^):	90	*R*_*p*_*(%):*	5.49
Direct cell volume $\left({Å}^{3}\right)$:	627.4744	*R*_*wp*_*(%):*	6.95
Density (g cm^−3^):	5.402	*R*_exp_*(%):*	5.40
		GoP:	1.66

Atom	*x*	*y*	*z*	B_iso_$\left({Å}^{2}\right)$	Occupancies
O1	0.38678	0.38678	0.38678	1.84499	0.97052
Fe2	0.00000	0.00000	0.00000	0.81581	0.97669
Zn3	0.62500	0.62500	0.62500	0.95477	0.51878
Fe4	0.62500	0.62500	0.62500	0.5477	0.98822
Cd5	0.00000	0.00000	0.00000	0.81581	0.99380
Cd6	0.62500	0.62500	0.62500	0.95477	0.95568
Amounts of elements (Weight %)					
Cd:	Zn:	Fe:		O:	
24.9	11.7	39.9		23.5	

The average crystallite size of the prepared sample of ZCFO was estimated using a modified form of the Scherrer formulation [46,47]:

(1) $$\ln\;\beta_{\text{hkl}}=\ln\;\left(\frac{K\lambda }{D}\right)+\ln\;\left(\frac{1}{\cos\;\theta }\right)$$

here, D represents the average crystallite size of the nanoparticles, K = 0.94 refers to the shape factor, ε refers to the microstrain, and the other symbols have the usual meanings. Fig. 1(c) shows the modified Scherrer plot, and the crystallite size of the ZCFO spinel ferrite nanoparticles estimated by using Eq. (1) was 51.37 nm. The average crystallite size of the sample was also calculated using Williamson-Hall (WH) formula [48]:

(2) $$\ln\;\beta_{\text{hkl}}=\frac{K\lambda }{D}+4\varepsilon \;\sin\;\theta$$

A typical WH plot is shown in Fig. 1(d). The calculated average crystallite size and microstrain, calculated in accordance with Eq. (2), were 49.62 nm and 0.000061, respectively, which are close to previously estimated values [49,50]. The X-ray density was estimated by the following formula [51,52]:

(3) $$\rho_{x}=\frac{Z.M}{V.N}$$

where Z is the number of molecules per unit cell (Z = 8), M is the molecular mass (M = 264.59 g/mol), V is the volume of the unit cell (V = a^3^ = 627.46 Å^3^) and N is the Avogadro number (6.022 × 10^23^ atoms/mole). The X-ray density of the sample calculated using Eq. (3) is 5.62 g/cm^3,^ which matches [5]. The density (ρ) of the prepared sample was also estimated by employing the famous Archimede method using the following equation [53]:

(4) $$\rho =\frac{W_{air}}{W_{air}-W_{water}}\times \rho_{water}$$

In the above equation, W_air_ and W_water_ are the weights of the sample measured in air and in water, respectively, whereas the room temperature density of water is 1 g/cm^3^. The measured density of the synthesized ZCFO sample Eq. (4) was 4.812 g/cm^3^. Moreover, the porosity (P) of the ZCFO sample was estimated Eq. (5) given as [54]:

(5) $$P=\frac{\rho_{x}-\rho }{\rho_{x}}\times 100\%$$

where $\rho_{x}$ and ρ represent the X-ray density and measured density of the ZCFO nanoparticles, respectively. The estimated porosity of the ZCFO nanoparticles sintered at 950 °C was 14.37 %. The dislocation density (δ) of the sample represents the number of imperfections and is calculated by the following relation: *δ* = *1/D*^*2*^ [55], where D represents the average crystallite size. The dislocation density of ZCFO was estimated to be 4.486 × 10^14^ m^−2^. A higher dislocation density indicates that the sample has a high mechanical hardness. The specific surface area (SSA) was calculated by the following equation [56]:

(6) $$SSA=\frac{600}{D\times \rho_{x}}\text{,}$$

where D is the average crystallite size and $\rho_{x}$ represents the X-ray density. The specific surface area is estimated using Eq. (6) comes out to be 22.614 m^2^/g.

**Fig. 2:**
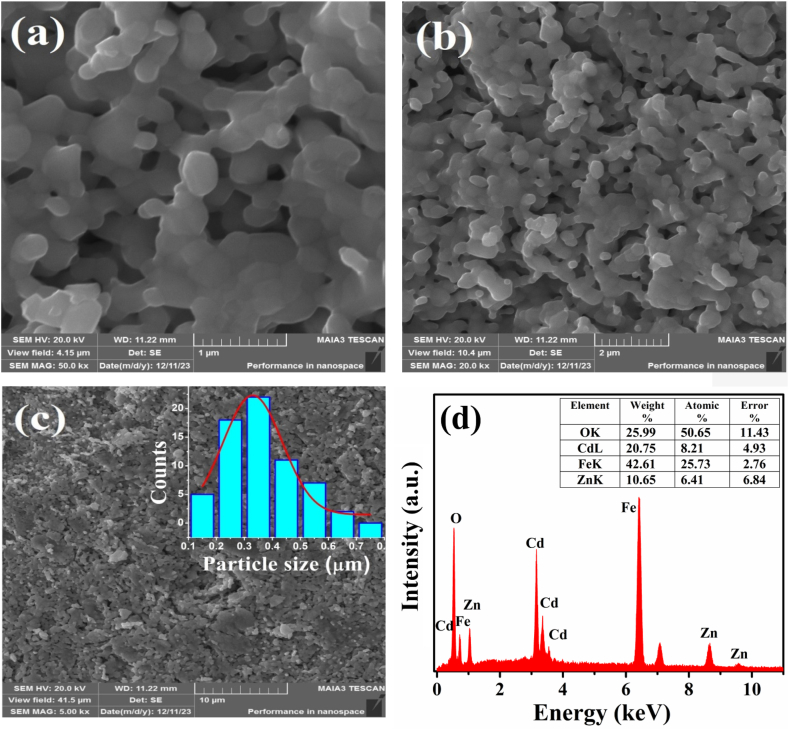
SEM micrographs of ZCFO spinel ferrite at magnifications of (a) 1 μm, (b) 2 μm and (c) 10 μm (the inset shows the particle size distribution), and (d) EDX spectrum of ZCFO spinel ferrite sintered at 950 °C.

Surface morphological analysis of the prepared ZCFO spinel ferrite was carried out using SEM. Fig. 2 shows micrographs of ZCFO for three different magnifications. The formation of spherical Zn_0.5_Cd_0.5_Fe_2_O_4_ spinel ferrite nanoparticles with a poly-dispersed distribution in the observed section of the sample was confirmed by SEM images. The agglomeration of the ZCFO nanoparticles can be clearly observed in the SEM images. This agglomeration in ferrites may be due to the magnetic interaction of dipole moments with each other. The average particle size of the prepared sample, estimated from SEM micrographs utilizing ImageJ software, was 329 nm. Fig. 2(d) shows the energy dispersive X-ray (EDX) spectrum of ZCFO spinel ferrite, confirming the presence of Zn, Cd, Fe and O as the constituent elements of the synthesized sample without any impurity atoms. The weight% and atomic percentage of each element present in the sample, along with the percent error, are depicted in the inset of Fig. 2(d) and are comparable to the weight percentages extracted from Rietveld analysis. The simplest ratio of the atomic percentages of the elements Zn:Cd:Fe:O was 0.5:0.64:2.01:3.95, which is consistent with the chemical formula Zn_0.5_Cd_0.64_Fe_2.01_O_3.95_. Hence, the EDX results verified the successful formation of the desired Zn_0.5_Cd_0.5_Fe_2_O_4_ as the major phase.

### 3.2 Impedance analysis

**Fig. 3:**
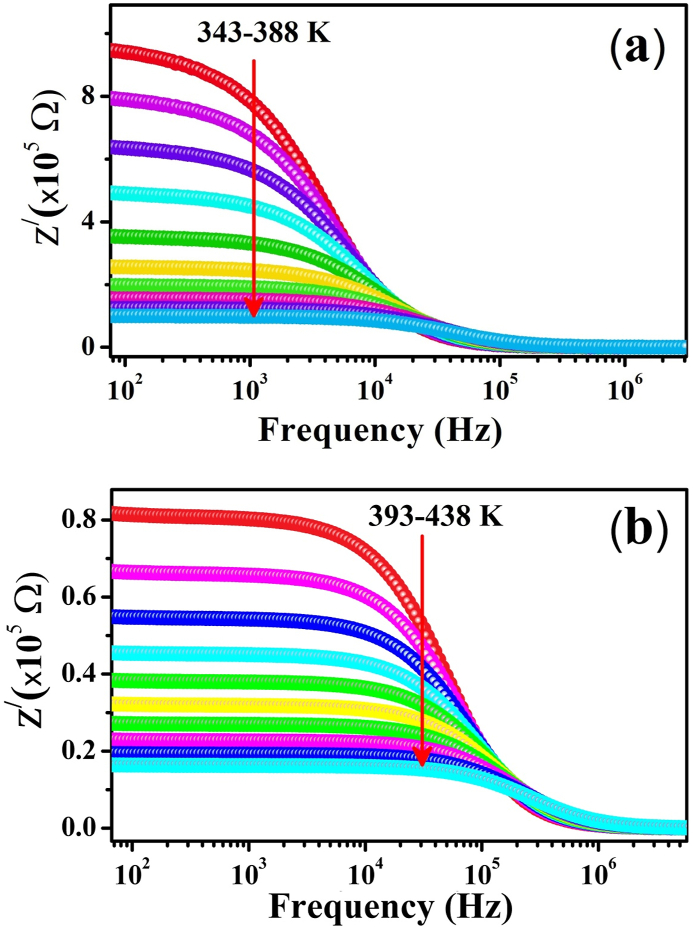
Frequency-dependent variation in the real part of the impedance Z^/^ of ZCFO spinel ferrite in the measured temperature ranges of (a) 343 K–388 K and (b) 393 K–438 K.

Complex impedance analysis is a very useful method for distinguishing microconstituents in which defects and vacancies may exist at electrode and grain interfaces or at grain boundaries [57]. Fig. 3(a and b) shows the plot between the real component (Z^/^) of the impedance of ZCFO spinel ferrite and the frequency in the temperature range of 343 K–438 K. The plot shows the scattering or dispersion of Z^/^ at lower frequencies for all temperatures. Z^/^ becomes unaffected above a specific frequency value of approximately 10^5^ Hz. This trend may indicate large conduction in the sample because of the existence of a hopping mechanism in the sample [58,59]. The region in the graph that is frequency dependent may be referred to as the reactive part, while the frequency-independent region of the graph signifies the resistive component of the material. The relaxation of neighboring charges to their equilibrium position occurs successfully in the low-frequency region, which results in long-range movements of charges, while in the region where the frequency is high, the relaxation of charge carriers to their position is due to the hopping of electrons, which results in the domination of short-range movements of charges in the higher-frequency region of the graph. The decreasing trend of the real part of the impedance (Z^/^) when the temperature increases indicates the existence of a negative temperature coefficient of resistance in the sample, which suggests the semiconducting behavior of ZCFO spinel ferrite [17]. The decreasing trend may be explained by the barrier to the motion of space charge, which decreases at higher frequencies and consequently may result in the possible release of space charge [60,61]. This behavior may also be responsible for the reduction in the resistive behavior of the material in the higher frequency region. The available time for the space charge to relax to its equilibrium position is too short in the higher frequency region, which results in a high recombination rate of charge carriers, indicating the release of space charge. This process is suggested when all lines merge towards the higher frequency region of real impedance for a given temperature range.

**Fig. 4:**
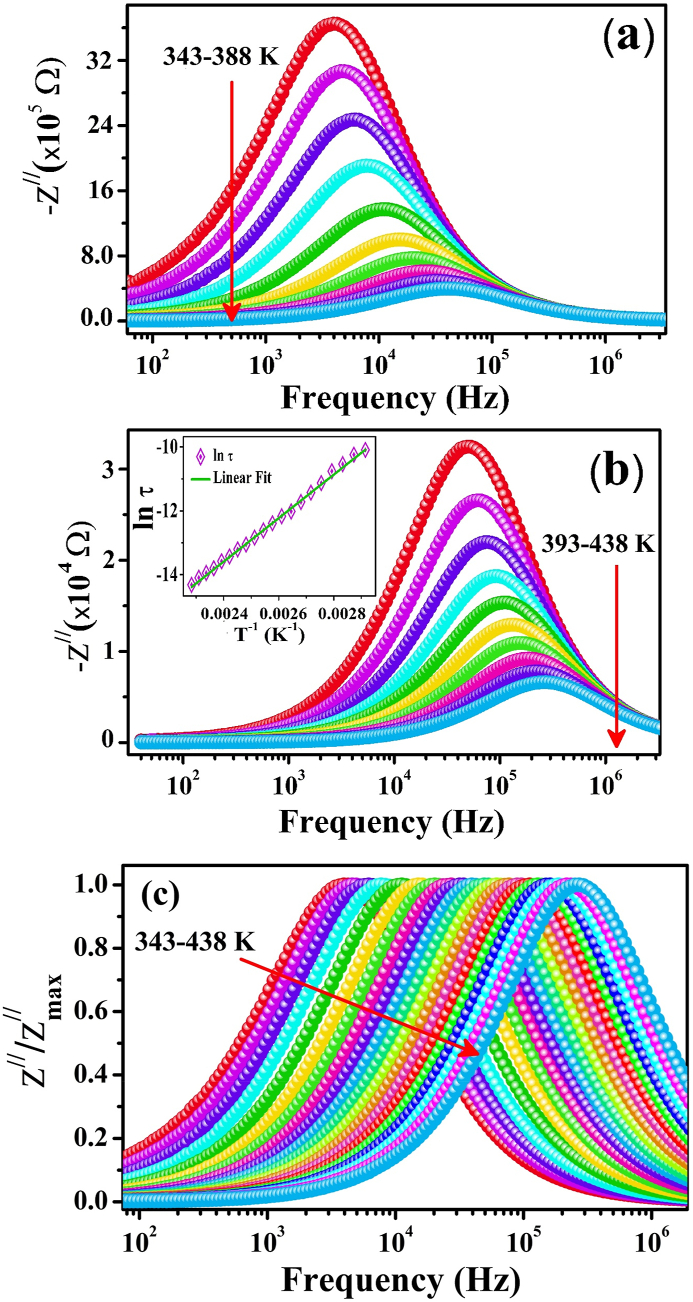
Frequency-dependent variation in the imaginary part of the impedance Z^//^ in the measured temperature ranges of (a) 343 K–388 K, (b) 393 K–438 K and (c) normalized spectra of Z^//^ of ZCFO spinel ferrite.

In Fig. 4(a and b), the graphs depict how the imaginary component (Z^//^) changes with AC frequency across the temperature ranges of 343 K–388 K and 393 K–438 K, respectively. A gradual increase in Z^//^ can be observed in Fig. 4 at lower frequencies until it reaches its maximum value $Z_{\max}^{//}$. This peak in the graph of Z^//^ against frequency indicates the presence of a relaxation mechanism in the sample [62]. The peak value of Z^//^ is referred to as the relaxation value, and each peak corresponds to a frequency known as the relaxation frequency (f_r_). After this relaxation frequency, a reduction in Z^//^ can be observed for a given temperature range and shows a constant trend at higher frequencies. This behavior of Z^//^ at higher frequencies may be assumed to be because the electric dipoles in the material are unable to respond to the high frequency of AC signals, leading to a decrease in polarization [61,63]. When the temperature increases, each peak decreases in height and translates towards higher frequencies. The height of the peak represents the resistive response of the material, so a decrease in the peak height suggests a decrease in the impedance due to an increase in the mobility of polarons caused by an increase in temperature [57]. This trend implies a decrease in relaxation time with increasing temperature, demonstrating that the sample undergoes a temperature-dependent space-charge relaxation process [62]. The trend of shifting relaxation peaks towards the high-frequency region with increasing temperature is in good agreement with the theoretical model [64]. The relaxation frequency corresponding to each temperature can be further used to extract the activation energy of relaxation using the Arrhenius-type relation given as:

(7) $$f_{\max}=f_{o}\;\exp\left(\frac{-E_{a}}{k_{B}T}\right)$$

where f_max_ represents the frequency related to the peak value of the imaginary part of the impedance Z^//^, T represents the absolute temperature, k_B_ represents the Boltzmann constant and f_o_ represents the pre-exponential frequency. The calculated value of the activation energy using Eq. (7) is 0.618 eV. In Fig. 4 (c), a normalized pattern of the ($Z^{//}/Z_{\max}^{//}$) imaginary component of impedance versus frequency is plotted in the temperature range from 343 K to 438 K, which shows the shifting of the peak height towards the high-frequency region with increasing temperature, indicating the distribution of relaxation times as a function of temperature. The peak frequency (f_max_) and relaxation time (τ) are described by the following equations:

(8) τ=1\/2πf_(max

The relaxation time calculated from Eq. (8) follows an Arrhenius-type relation, and the activation energy of the relaxation process, E_a_, was extracted from the relation:

(9) $$\tau =\tau_{o}\;\exp\left(\frac{-E_{a}}{k_{B}T}\right)$$

where “τ_o_” denotes the preexponential factor. The activation energy of the relaxation process was estimated from the slope of the linear fit of the graph between 1/T and ln τ, as shown in the inset of Fig. 4(b). The value calculated for E_a_ from the above expression (Eq. (9)) was 0.606 eV, confirming the existence of a hopping phenomenon in ZCFO spinel ferrite, which was activated thermally.

**Fig. 5:**
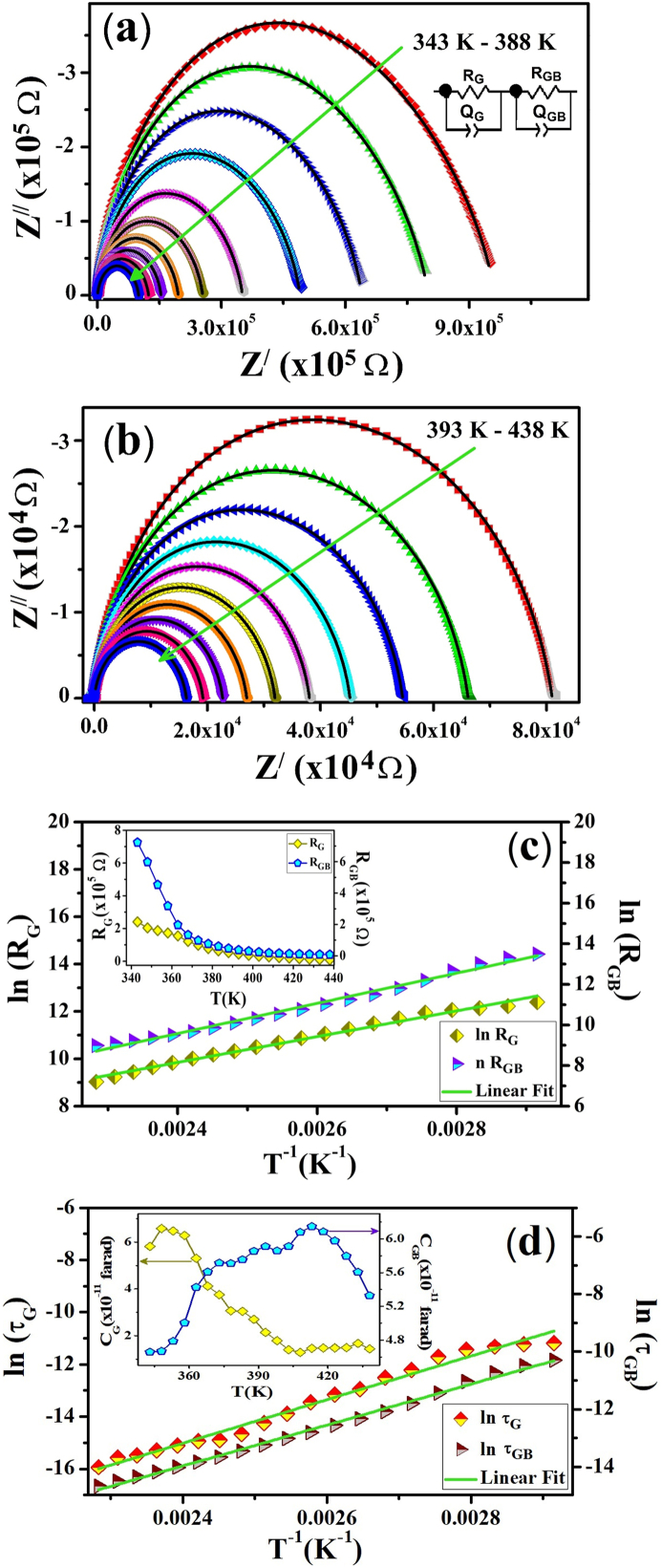
Equivalent circuit model-fitted Nyquist plots of ZCFO spinel ferrite in the measured temperature ranges of (a) 343 K–388 K and (b) 393 K–438 K and temperature-dependent variations in the (c) resistance and (d) relaxation times (the inset shows the temperature evolution of the grain and grain-boundary capacitances).

Fig. 5(a) and (b) show the equivalent circuit model-fitted Nyquist plots for ZCFO spinel ferrite in the experimental temperature ranges of 343 K–388 K and 393 K–438 K, respectively. The frequency is maximal near the origin, and it decreases from the left to the right end of every spectrum. At all temperatures, no linear portion is evident in the lower frequency region. Moreover, no shift along the real axis (away from the origin) in the semicircles is observed at any measured temperature. These observations suggest that the electrode-sample interface resistance and/or electrode polarization are either not present or low enough to contribute to the net or overall impedance of the ZCFO spinel ferrite sample [45]. Each semicircle in the graph is a combination of two depressed semicircles, and their centers lie below the real axis. These two semicircular arcs suggest the contributions of grain boundaries (interfacial) and grains (bulk) to the overall electrical response of the sample.

The diameter of all semicircular arcs decreases as the temperature increases, suggesting that the overall resistance of the sample is inversely related to the temperature. Thus, it can be concluded that the capacitive and resistive components of the sample vary with temperature, suggesting that the process is thermally activated. To explain how the sample responds when it is subjected to a sinusoidal AC signal, an electrical circuit was suggested by fitting the experimentally observed data with an equivalent circuit model. In Fig. 5(a) and (b), the observed data are depicted by coloured symbols, whereas the black solid lines correspond to the fitted data. The fitting of the equivalent circuit model to the experimental data was performed using ZView software. In the inset of Fig. 5(a), the proposed equivalent circuit model is shown, which consists of two parallel combinations connected in series expressed as (R_G_Q_G_) (R_GB_Q_GB_), where R_G_ and R_GB_ represent the resistances of the grain and grain boundaries, respectively, and Q_G_ and Q_GB_ label the constant phase elements for the grain and grain boundary interfaces, respectively. Various parameters extracted from Nyquist plot fitting are summarized in Table 2. In general, the inclusion of a constant phase element, CPE (Q), manipulates the non-ideal behaviors of resistive and capacitive responses from interfacial (grain-boundaries) and bulk (grains) components of the material. The impedance Z(ω) corresponding to the CPE is calculated by the relation [65]:

(10) $$Z\left(\omega \right)=\frac{1}{Q{\left(i\omega \right)}^{n}}=\frac{1}{Q{\left(\omega \right)}^{n}}\left[\cos\;\left(\frac{n\pi }{2}\right)-i\;\sin\;\left(\frac{n\pi }{2}\right)\right]\text{,}$$

where $i=\sqrt{-1}$, ω and Q label the angular frequency and the capacitance corresponding to the CPE, respectively. The parameter ‘n’ in Eq. (10), infers the non-ideal response of the material and attains a value in the range 0≤n≤1. The capacitance offered by the grains and grain boundaries was evaluated from the Q values using the relation $C=R^{1-n}Q^{1/n}$.

**Table 2:** Electrical parameters extracted from the Nyquist plots.

T(K)	R_G_ ($10^{4}\;\Omega$)	Q_G_ (nF)	$\alpha_{G}$	C_G_ ($10^{-11}$ F)	R_GB_ ($10^{4}\;\Omega$)	Q_GB_ ($10^{-10}$ F)	$\alpha_{GB}$	C_GB_ ($10^{-11}$ F)
343	24.1	4.89	0.77108	5.81	72.2	1.18	0.90936	4.66
353	18.7	2.88	0.79163	6.47	45.3	1.19	0.91532	4.79
363	15.4	1.18	0.82047	5.31	19.7	1.19	0.9312	5.43
373	9.77	0.91	0.83237	3.75	9.79	1.19	0.93952	5.71
383	6.33	0.83	0.84046	3.05	6.04	1.20	0.94178	5.75
393	4.37	0.72	0.84985	2.14	3.73	1.19	0.94625	5.91
403	3.03	0.66	0.85729	1.41	2.41	1.10	0.9536	5.91
413	2.22	0.62	0.85905	1.46	1.58	1.14	0.95516	6.15
423	1.53	0.65	0.85977	1.48	1.16	1.18	0.9522	5.98
433	1.02	0.72	0.86187	1.67	0.89	1.24	0.94521	5.61

The values of resistances obtained from fitting the equivalent circuit for grains and grain boundaries are plotted as a function of frequency and expressed in the inset of Fig. 5(c), where it can be observed that the resistances of both the grain boundaries and grains decrease as the temperature increases, suggesting that ZCFO spinel ferrite has semiconducting behavior to input the AC signal. These results are in good agreement with the results obtained from the Z^/^ and Z^//^ analyses. Furthermore, it can be observed from the graph that at all temperatures, the resistance offered by grains is lower than the resistance offered by grain boundaries. This trend may indicate that grain boundaries contain lower concentrations of defects and trapped charges than grains because the potential barrier offered by the greater defect concentration region is low for charge carriers, which are thermally activated [66]. Similar patterns have been reported by Muhammad Javaid et al*.* [61] for sol gel-derived NiCr_2_O_4_ spinel chromite and Rahmouni et al*.* [67] for 20 % Ti-doped La_0.7_Sr_0.3_MnO_3_ perovskite. The changes in the capacitance of the grains (C_G_) and the capacitance of the grain boundaries (C_GB_) with temperature are represented in the inset of Fig. 5(d). An overall decreasing trend of C_G_ with temperature is observed, while C_GB_ increases with increasing temperature, but above 400 K, it decreases again. This decrease in the C_G_ curve with increasing temperature can be attributed to the release of charges (which were trapped) by the gain of enough energy to cross the potential barrier by the hopping mechanism, while the increase in temperature may be responsible for the accumulation of charges on the grain boundaries, which results in an increasing pattern of C_GB_ as the temperature increases [68]. The values of the resistances corresponding to the grains and grain boundaries obtained from the equivalent circuit model were fitted with the following Arrhenius equation to evaluate the activation energy [69]:

(11) $$R=R_{o}\;\exp\;\left(\frac{-E_{a}}{k_{B}T}\right)$$

where R_o_ is the preexponential factor and the other symbols have the usual meanings. The activation energy, E_a_, calculated for conduction through grains is 0.4693 eV, and its value for conduction through grain boundaries is 0.6414 eV, as obtained by fitting Eq. (11), as shown in Fig. 5(c). For the calculation of relaxation times, the equation τ=RC was applied, where R represents the bulk and interfacial resistances and C represents the capacitance, which were obtained by fitting the Nyquist plot. The E_a_ for the relaxation mechanism was estimated by the Arrhenius equation $\tau =\tau_{o}\;\exp\;\left(\frac{-E_{a}}{k_{B}T}\right)$. The value of E_a_ for the relaxation mechanism calculated for grains is 0.7208 eV, and its value for grain boundaries is 0.6132 eV. These activation energies were calculated from the slopes of the ln τ vs T^−1^ graphs, as depicted in Fig. 5(d).

### 3.3 Conductivity analysis

**Fig. 6:**
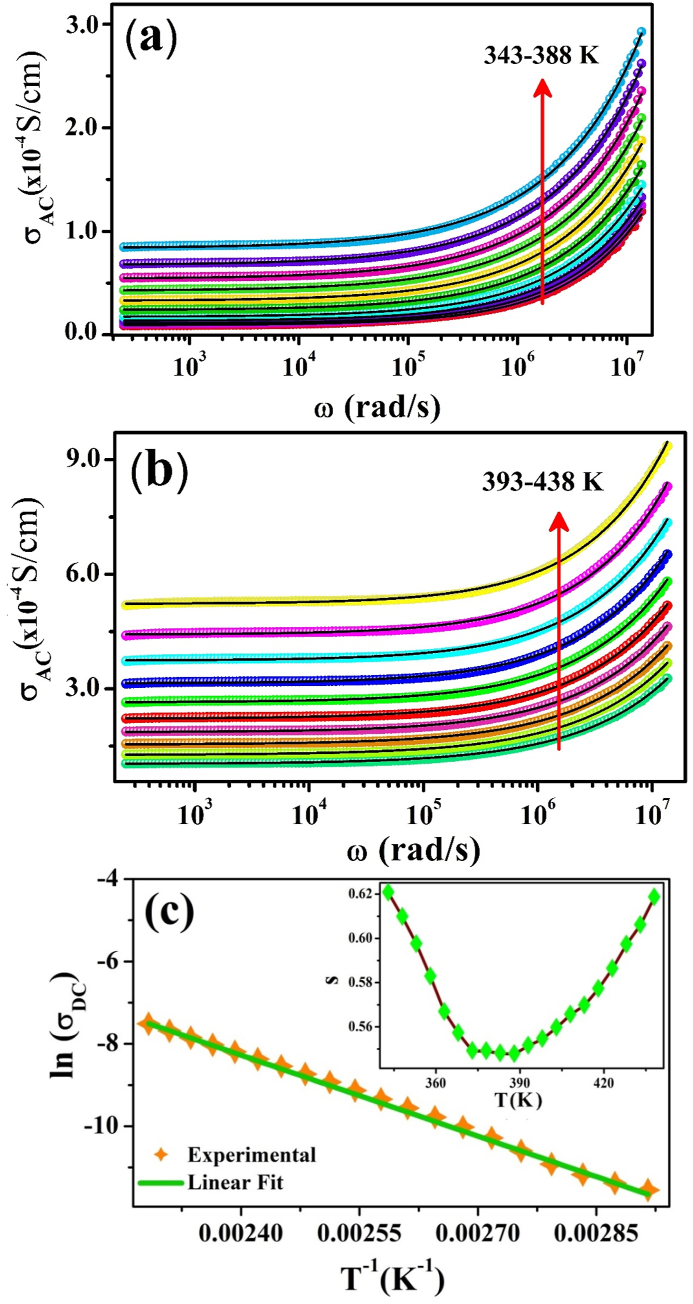
AC conductivity variation as a function of angular frequency in the measured temperature ranges of (a) 343 K–388 K, (b) 393 K–438 K, and (c) variation in DC conductivity with temperature for ZCFO spinel ferrite (the inset shows the temperature dependence of the s parameter).

The change in the electrical conductivity (σ_AC_) of ZCFO as a function of frequency in the temperature range of 343 K–438 K is represented in Fig. 6(a and b). It can be observed from the conductivity curves that there is no change in conductivity at lower frequencies, suggesting the existence of DC conductivity, which may be ascribed to the long-range motion of charge carriers in response to the applied sinusoidal electric field. Above the intermediate frequency, there is an increasing trend in the AC conductivity. This dispersion in conductivity shows the contributions to the total conduction from both reactive and resistive components of the sample. The AC conductivity of the sample may be attributed to a relaxation mechanism due to the hopping of charge carriers. The overall AC conductivity of the sample is the sum of both the DC and AC conductivities, as represented by Jonscher's power law equation [70]:

(12) $$\sigma_{total}=\sigma_{DC}+A\omega^{s}$$

where $\sigma_{DC}$ represents the DC part of the conductivity of the sample and $A\omega^{s}$ is the AC part of the conductivity. The term Aω^s^ is attributed to the frequency-dependent AC part of the conductivity, and the exponent s (0 ≤ s ≤ 1) is a temperature-dependent parameter that signifies the long-range dynamics of charge carriers. In Fig. 6(a and b), the experimental conductivity data (shown by coloured symbols) were fitted with Jonscher's power law (shown by black solid lines) in accordance with Eq. (12). The parameter “s” and its dependence on temperature determine the type of charge transport mechanism responsible for the conduction process in the material [[71], [72], [73], [74]]. Many theoretical mechanisms of hopping models have been proposed on the basis of the temperature-dependent coefficient s(T), by which the conduction mechanism in the sample can be explained. In the correlated barrier hopping (CBH) process, “s” has an inverse relation with temperature [71], whereas in small polaron hopping (SPH) phenomena, “s” has a direct relation with temperature [72], and if “s” remains nearly equal to 0.8 or increases slightly as temperature increases or exhibits temperature-independent behavior, then the conduction mechanism refers to the quantum mechanical tunneling (QMT) process [73]. In the overlapping large polaron hopping (OLPH) mechanism, the value of “s” initially decreases to a minimum value and then increases [74]. The variation in the s parameter with temperature in the current study is displayed in the inset of Fig. 6(c). Initially, s decreases, reaches a minimum and then increases, which suggests that the overlapping large polaron hopping (OLPH) model is responsible for the conduction mechanism in ZCFO spinel ferrite. In the current study, there are two different regions in the conductivity graph of ZCFO: (a) the conductivity spectrum, which is independent of frequency in the low-frequency region and refers to the DC conduction process, and (b) the frequency-dependent region of the graph, which refers to AC conduction through grains at higher frequencies.

The change in DC conductivity of the sample with temperature represented in Fig. 6(c) is observed to follow the Arrhenius relation:

(13) $$\sigma_{DC}=\sigma_{o}\;\exp\;\left(\frac{-E_{a}}{k_{B}T}\right)$$

where σo represents the preexponential factor and E_a_ indicates the activation energy of conduction. The linear fitting of the graph between ln σ_DC_ and T^−1^ gives the activation energy “Ea” for DC conduction, as shown in Fig. 6(c). The activation energy calculated for DC conduction from Eq. (13) is 0.566 eV.

### 3.4 Electrical modulus analysis

Complex electric modulus analysis is a very useful method for analysing the electrical behaviour of samples with the same resistance but different capacitance [75]. At the electrode sample interface, conduction through grain boundaries and grains may be responsible for the polarization effect in the sample. Using complex modulus formalism, the process of relaxation can be investigated by observing the electric behavior of the sample when it is placed in a sinusoidal electric field. The complex electric modulus (M*) has two components, the real part of the modulus (M^/^) and the imaginary part of (M^//^), which are estimated by Eqs. (14), (15), (16), given as:

(14) $$M*=\frac{1}{\varepsilon *}$$

(15) $$M*=M^{/}+iM^{//}$$

(16) $$M*=\frac{\varepsilon^{/}}{{\varepsilon^{/}}^{2}+{\varepsilon^{//}}^{2}}+i\frac{\varepsilon^{//}}{{\varepsilon^{/}}^{2}+{\varepsilon^{//}}^{2}}$$

**Fig. 7:**
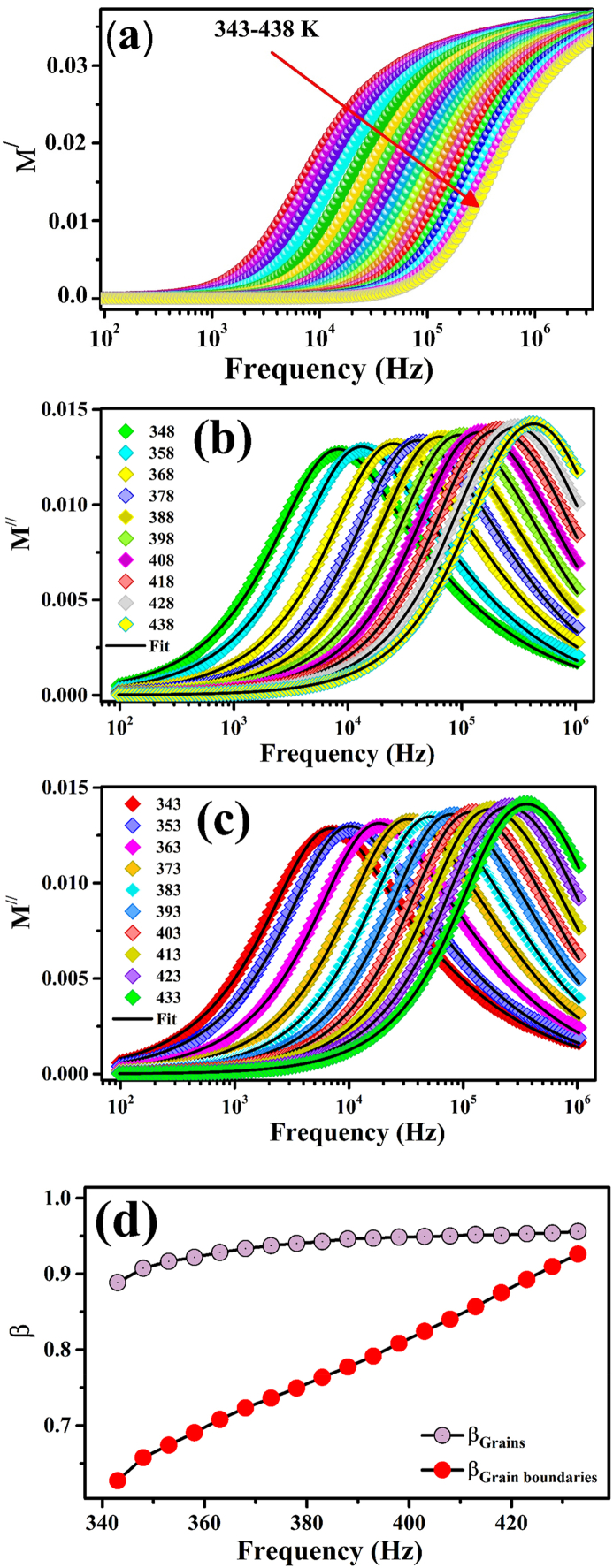
Frequency-dependent variations in the (a) real part M^/^ and (b–c) imaginary part M^//^ of the electric modulus (the symbols represent the experimental data, while the black solid lines correspond to the fit of the KWW equation) and (d) the temperature evolution of the stretched coefficient for ZCFO spinel ferrite in the measured temperature range from 343 K to 438 K.

The changes in M^/^ and M^//^ of the electric modulus of ZCFO spinel ferrite as a function of frequency in the applied temperature range of 343 K–438 K are shown in Fig. 7(a) and (b), respectively. In the lower frequency region, M^/^ exhibits significantly low values approaching zero. These small values of M^/^ in the low-frequency region may suggest the absence of electronic polarization, which is responsible for the long-range movements of charges [70]. However, at lower frequencies, electrode sample polarization may occur; however, the contribution of this polarization is negligible because of the absence of a restoring force, which governs the motion of the charges under the influence of the applied electric field [76]. As the applied frequency increases, the contributions to the conduction process increase due to the short-range mobility of charge carriers, and the magnitude of the real part (M^/^) of the modulus increases with increasing frequency and reaches a saturation stage at all temperatures. Therefore, the saturation of M^/^ suggests that the short-range movements of charges are responsible for the relaxation phenomena in the sample [77]. Furthermore, the real part (M^/^) decreases with increasing temperature. This trend of M^/^ may be attributed to the existence of a thermally activated relaxation mechanism because at higher temperatures, the alignment of dipoles becomes quite easy in the ZCFO spinel ferrite sample.

In Fig. 7(b), the imaginary component of the electric modulus (M^//^) is plotted versus the frequency in the applied temperature range of 343 K–438 K. Initially, M^//^ increases with increasing frequency and reaches a maximum value at a specific frequency, known as the relaxation frequency (f_max_); afterwards, it decreases with increasing frequency at all temperatures. All curves of the imaginary component (M^//^) of the electric modulus may be distributed into three different regions. The first region, which is below the relaxation frequency value (f_max_), may be ascribed to long-range movements of charges. In this region, the charge carriers may exhibit a long-range hopping process, i.e., they can hop easily from one lattice position to neighboring lattice positions [78]. The second region at higher frequencies, which exists above the relaxation frequency, may be ascribed to short-range movements of charge carriers inside a potential well [79]. The third region of the graph is the peak of the imaginary component (M^//^) and is associated with the transition region of the charge carriers from long-range mobility to short-range motion. The asymmetric pattern and broadening of the peak below and above f_max_ suggest the non-Debye type of relaxation process in the ZCFO spinel ferrite. The shift in the peak towards the high-frequency region with increasing temperature suggests the existence of a distribution in the relaxation time as the temperature increases. Fig. 7(b) illustrates the increase in the height of the peak as a function of temperature, suggesting a decrease in the capacitance of the material, as the capacitance is inversely related to the peak height. The dielectric relaxation characteristics of electroactive ceramics can be further explored by the Kohlrausch-Williams-Watts (KWW) method. The time dependence of the electric field is described by the equation:

(17) $$E\left(t\right)=\exp\;\left[-{\left(\frac{t}{\tau_{\max}}\right)}^{\beta }\right]\text{.}$$

where $\tau_{\max}$ is the relaxation time corresponding to the conduction process and β denotes the full width at half maximum (FWHM) of the relaxation peak with a magnitude in the range 0≤β≤1. The variation in the magnitude of exponent β in Eq. (17), describes the degree of deviation from the ideal Debye-type relaxation in the system. The frequency-dependent behavior of the complex modulus is well described by this decay function by employing the function given as [80]:

(18) $$M*\left(\omega \right)=M_{\infty }-M_{\infty }\left[\frac{1}{\exp\left(i\omega t\right)}\left(-\frac{dE\left(t\right)}{dt}\right)dt\right]$$

In the present work, the frequency-dependent imaginary part of the modulus of ZCFO spinel ferrite in the measured temperature range was fitted with the modified KWW relation to elucidate the relaxation mechanism. The modified form of imaginary part of modulus corresponding to Eq. (18), proposed by Bergman is given as [81]:

(19) $$M^{//}\left(\omega \right)=\sum_{j=1}^{2}\frac{M_{j\;\max}^{\backslash //}}{\left(1-\beta_{j}\right)+\left(\frac{\beta_{j}}{1+\beta_{j}}\right)\left[\beta_{j}{\left(\frac{\omega_{j\;\max}}{\omega }+\frac{\omega }{\omega_{j\;\max}}\right)}^{\beta_{j}}\right]}\text{.}$$

where $M_{j\;\max}^{//}$ refers to the maximum relaxation peak, $\omega_{j}$ is the relaxation frequency, and $\beta_{j}$ is the stretched coefficient. The stretch coefficient β determines the nature of the relaxation process. A value of unity implies that the relaxation process is of the ideal Debye type, whereas a value less than unity infers deviation from the ideal Debye type relaxation. For an ideal dielectric, interactions between dipoles are negligible, leading to Debye-type relaxation in the material. Therefore, a value of β smaller than unity can be ascribed to increased dipole‒dipole interactions [[82], [83], [84]]. The fitted profiles of the imaginary modulus with Eq. (19) over the measured temperature range for ZCFO spinel ferrite are depicted in Fig. 7(b and c), where good agreement between the experimental data and fitted profile is evident. The temperature evolution of the stretched coefficient β is depicted in Fig. 7(d), where an increasing trend of β with temperature can be observed for both grains and grain boundaries. Moreover, β was less than unity for both grains and grain boundaries, signifying non-Debye-type relaxation in both electroactive regions of the sample. Moreover, larger values of β for grain boundaries than for grains can be related to increased dipole‒dipole interactions in grain boundary regions, which slow the relaxation of dipoles; hence, the relaxation frequency also decreases [54,85,86].

**Fig. 8:**
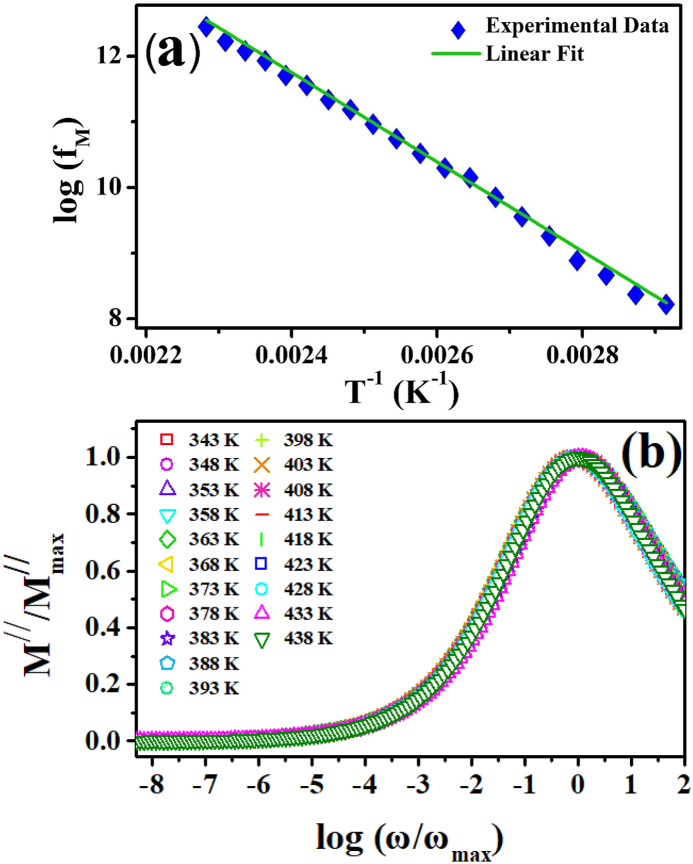
(a) Temperature dependence of the relaxation frequency and (b) scaling behavior of the imaginary modulus of ZCFO spinel ferrite in the measured temperature range of 343 K–438 K.

The variation in f_max_ with the inverse of temperature is represented in Fig. 8(a), which may be mathematically expressed using the Arrhenius relation:

(20) $$f_{\max}=f_{o}\;\exp\;\left(\frac{-E_{m}}{k_{B}T}\right)$$

In Eq. (20), f_o_ represents the pre-exponential factor, and E_m_ is the activation energy of the relaxation phenomenon. Fitting Eq. (20) with the graph shown in Fig. 8(a) gives the activation energy “E_m_” estimated from the electrical modulus analysis. The calculated value of “Em” (0.621 eV) is close to the value of the activation energy calculated from the impedance data. This observation confirms the existence of a thermally activated hopping mechanism, which is responsible for the charge transport process in ZCFO spinel ferrite [87]. The activation energies extracted for relaxation from Z^//^ of impedance analysis and electrical modulus analysis are in good agreement. This may be attributed to the capacitive response of the sample under the applied AC signal.

The plot between the normalized modulus and the normalized angular frequency in the varying temperature range of 343 K–438 K depicting the scaling behavior of the ZCFO spinel ferrite is shown in Fig. 8(b). All the curves in the graph exhibit a frequency-independent response in the low-frequency region, suggesting long-range movements of charges. Then, each curve reaches its peak value, suggesting that the transition region corresponds to the transition of the long-range mobility of charge carriers to short-range mobility. The short-range movements of charges suggest the localized motion of charges inside the potential well when subjected to a high-frequency applied electric field. Furthermore, all curves in the graph merge in a given temperature range at all frequencies, suggesting the occurrence of temperature-independent relaxation phenomena during the conduction process in the sample and that the number of charges changes due to variations in the temperature, but the overall conduction process in the material remains the same [88].

### 3.5 Dielectric analysis

The complex permittivity (ε*) can be expressed in terms of the real ($\varepsilon^{/}$) and imaginary ($\varepsilon^{//}$) components of the permittivity as:

(21) $$\varepsilon *=\varepsilon^{/}-i\varepsilon^{//},\left|\varepsilon *\right|=\sqrt{{\left(\varepsilon^{/}\right)}^{2}+{\left(\varepsilon^{//}\right)}^{2}}$$

**Fig. 9:**
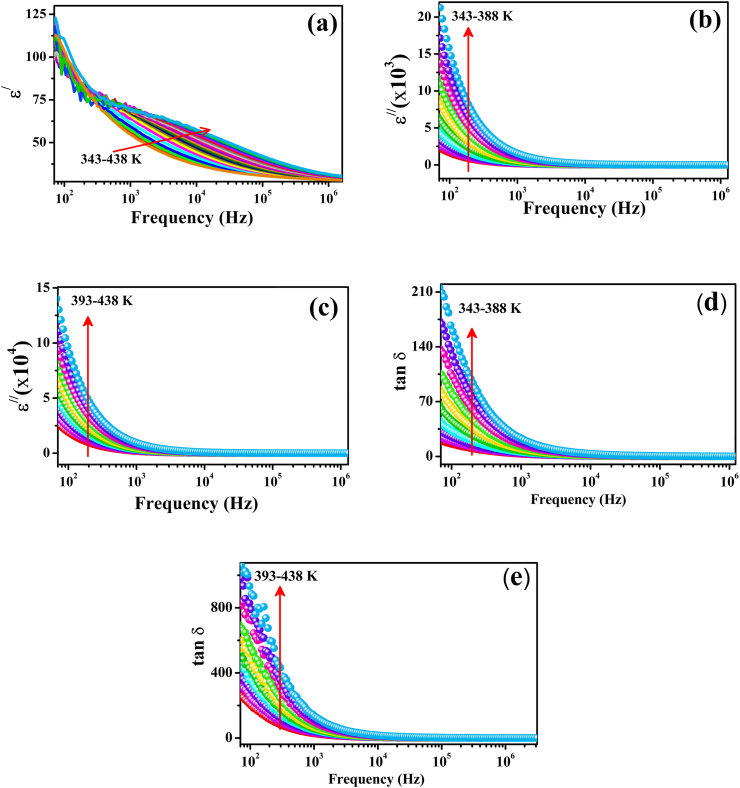
Frequency-dependent variations in the (a) real part of the dielectric constant, (b–c) imaginary part of the dielectric constant, and (d–e) dielectric tangent loss of ZCFO spinel ferrite in the measured temperature range of 343–438 K.

**Fig. 10:**
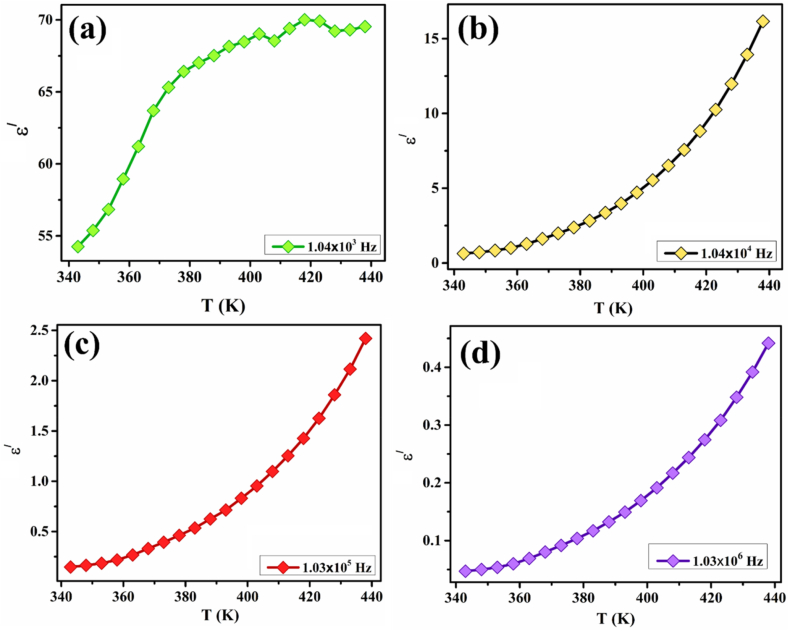
Temperature-dependent variation in the real part of the dielectric constant at various frequencies in the measured temperature range of 343–438 K for ZCFO spinel ferrite.

**Fig. 11:**
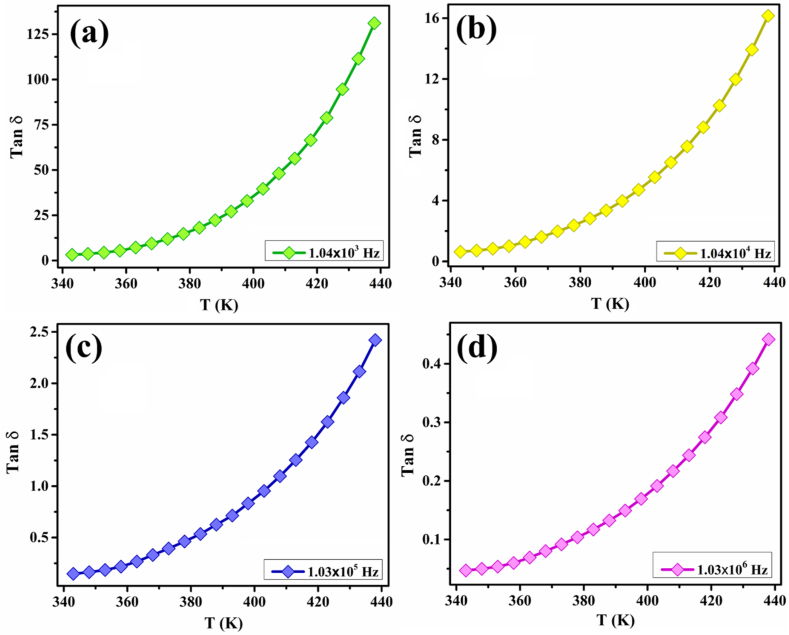
Temperature-dependent variation in tan loss at various frequencies in the measured temperature range of 343 K–438 K for ZCFO spinel ferrite.

The real and imaginary parts of the dielectric permittivity presented in Eq. (21), along with the tangent loss were calculated from the impedance data using the following equations [89]:

(22) $$\varepsilon^{/}=\frac{-Z^{//}t}{A\omega \varepsilon_{0}\left[{Z^{/}}^{2}+{Z^{//}}^{2}\right]}$$

(23) $$\varepsilon^{/}=\frac{Z^{/}t}{A\omega \varepsilon_{0}\left[{Z^{/}}^{2}+{Z^{//}}^{2}\right]}$$

(24) $$\tan\;\delta =\frac{\varepsilon^{//}}{\varepsilon^{/}}$$

where ω = 2πf represents the angular frequency of the input AC signals and A and t label the area and thickness of the prepared pellet, respectively. Fig. 9(a–c) depicts the variation in $\varepsilon^{/}$ and $\varepsilon^{//}$ of ZCFO spinel ferrite, calculated using Eqs. (22), (23)), as a function of frequency at temperatures ranging from 343 K to 438 K. It is worth noting that $\varepsilon^{/}$ infers the energy stored in the material, whereas $\varepsilon^{//}$ corresponds to the energy dissipated within the sample during different electrical processes. At all temperatures, high values of permittivity were observed at lower frequencies. A sudden reduction in the permittivity was observed with increasing frequency, and each curve reached an almost constant, frequency-independent value at all temperatures. The constant behavior of the dielectric permittivity with frequency may be attributed to the presence of charges and vacancies that are not able to respond to an applied electric field at higher frequencies [90]. At higher frequencies, grain boundaries respond less effectively, which allows grains to behave more effectively, as the hopping mechanism decreases at high frequencies of the AC input signal, causing a decrease in the dielectric permittivity at higher frequencies [90]. Furthermore, the sudden decrease in dielectric permittivity at lower frequencies may have emerged from deformational effects in the sample because of the movement of ions and electrons, and the relaxation mechanism depends upon the dipole orientation. As the frequency increases, the orientation polarization in the sample decreases, and molecular dipoles in the material take a long time to change their alignment, which is therefore responsible for the sharp reduction in the dielectric permittivity [91,92]. In the high-frequency region, the polarization due to the orientation of dipoles is stopped, so the dielectric permittivity exhibits a constant pattern, suggesting contact-sample interface polarization [93]. It can be observed from the graph that the real and imaginary parts of the dielectric permittivity increase with increasing temperature. The variation in the real and imaginary parts of the dielectric constant as a function of temperature at frequencies of 1.04 kHz, 10.4 kHz, 103 kHz and 1030 kHz is shown in Fig. 10(a–d) and Fig. 11(a–d), and summarized in Table 3, Table 4, at selected temperatures. An increase in the real part of the dielectric constant can be observed at all selected frequencies. This trend can be explained by the dipole orientation exhibiting a direct relation with temperature, so the dielectric permittivity increases with increasing temperature [94,95]. At lower temperatures, the molecular dipoles have very low magnitudes of thermal motion, so they cannot position themselves under an applied electric field. An increase in the temperature causes an increase in the thermal vibrations of molecular dipoles, facilitating their position in the direction of the electric field, so the polarization due to the orientation of dipoles increases, which is responsible for the large dielectric permittivity [96].

**Table 3:** Real part of the dielectric constant at selected temperatures and frequencies for ZCFO ferrite sintered at 950 °C.

Frequency (Hz)	343 K	353 K	363 K	373 K	383 K	393 K	403 K	413 K	423 K	433 K
2 × 10^2^	81.7	80.5	81.5	83.8	83.2	82.0	80.1	81.2	81.5	79.9
2 × 10^3^	47.5	50.0	54.2	58.7	61.0	62.6	63.7	65.2	65.8	66.2
2 × 10^4^	34.4	35.4	37.5	39.9	42.2	44.4	46.5	48.4	50.3	52.1
2 × 10^5^	29.7	29.9	30.4	31.1	31.9	32.8	33.8	34.8	36.0	37.4
2 × 10^6^	27.8	27.9	28.0	28.1	28.3	28.6	28.8	28.9	29.0	29.7

**Table 4:** Imaginary part of the dielectric constant at selected temperatures and frequencies for ZCFO ferrite sintered at 950 °C.

Frequency (Hz)	343 K	353 K	363 K	373 K	383 K	393 K	403 K	413 K	423 K	433 K
2 × 10^2^	817.4	1208.4	2172.1	3880.9	6126.3	9351.1	13881.2	19875.2	28040.1	39319.1
2 × 10^3^	94.2	134.1	232.3	404.7	630.2	953.8	1408.2	2012.0	2833.8	3971.9
2 × 10^4^	13.9	18.8	30.0	48.6	72.1	105.1	151.1	212.1	294.8	409.4
2 × 10^5^	3.0	3.7	5.3	7.8	10.8	14.7	20.0	26.8	35.8	47.9
2 × 10^6^	1.0	1.1	1.4	1.8	2.2	2.8	3.5	4.5	5.7	7.2

The change in tangent loss (tan δ) as a function of frequency in the temperature range of 343–438 K, calculated using Eq. (24), is represented in Fig. 9(d, e), and a summary of tangent loss values at various frequencies and temperatures is presented in Table 5. It can be observed from the graph that there is dispersion in the tan loss in the low-frequency region. The tan loss decreases with increasing frequency in the considered temperature range. This trend may imply the existence of a thermally activated relaxation mechanism of charges in the material, and the transport process is administered by hopping of charge carriers because in the conduction process exhibiting hopping phenomena, peak loss in the graph is considered to be a crucial factor [73,97]. The tan loss is observed to increase with increasing temperature, as depicted in Fig. 11. In the lower temperature range, the conductivity of the sample is low, so the tan loss is also minimal. However, when the temperature increases, the conductivity of the material increases, which may be responsible for the increase in loss during conduction, so it can be interpreted that the tan loss is directly related to temperature [98,99]. The temperature-dependent response of tan loss also confirms the occurrence of a thermally activated hopping process in the sample. All these results are in good agreement with the conductivity, impedance and modulus results reported earlier.

**Table 5:** Tan (δ) at selected temperatures and frequencies for ZCFO ferrite sintered at 950 °C.

Frequency (Hz)	343 K	353 K	363 K	373 K	383 K	393 K	403 K	413 K	423 K	433 K
2 × 10^2^	10.0	15.0	26.6	46.3	73.6	113.9	173.2	244.7	344.1	491.5
2 × 10^3^	1.98	2.67	4.28	6.88	10.32	15.23	22.09	30.83	43.03	60.12
2 × 10^4^	0.40	0.53	0.80	1.21	1.70	2.36	3.24	4.37	5.86	7.85
2 × 10^5^	0.10	0.12	0.17	0.25	0.33	0.44	0.59	0.77	0.99	1.28
2 × 10^6^	0.03	0.04	0.05	0.064	0.07	0.09	0.12	0.15	0.19	0.24

It is worth noting that the studied sample is not composed entirely of a single phase but rather of a major phase Zn_0.5_Cd_0.5_Fe_2_O_4_ (95.9 %) and a minor phase CdO (4.1 %), as determined from Rietveld refinement. However, complex impedance analysis revealed a single relaxation process, as evidenced by the single peak in each frequency-dependent spectrum of the imaginary impedance and modulus. Moreover, the Nyquist plot analysis suggested contributions only from grains and grain boundaries, with no or negligible contributions from electrodes or impurity phases. Thus, one can safely assume that the electrical and dielectric properties reported in this work are predominantly governed by the Zn_0.5_Cd_0.5_Fe_2_O_4_ spinel phase. However, future optimization and improvement of the synthesis protocol and electrical and dielectric studies of ZCFO spinel ferrite over a wider range of temperatures and frequencies can significantly contribute to the current understanding of the electrical transport properties of the studied material.

## 4 Conclusion

In the present study, Zn_0.5_Cd_0.5_Fe_2_O_4_ spinel ferrite was synthesized via a sol-gel combustion method. The phase identification and structural parameters of the synthesized material, which was sintered at 950 °C for 4 h, were studied via XRD analysis. The Rietveld refinement results confirmed the formation of the Zn_0.5_Cd_0.5_Fe_2_O_4_ spinel ferrite phase with space group $Fd\bar{3}m\text{.}$ The crystallite sizes estimated using the modified Scherrer formula and the WH plot were 51.37 nm and 49.62 nm, respectively. SEM analysis of the surface morphology revealed that the grains and grain boundaries were the microconstituents of the sample. The distribution of the relaxation time and translation of the peak in the impedance data and the information extracted from the electric modulus analysis confirmed that the thermally activated hopping mechanism was the dominant phenomenon for the electric charge transport process in the sample. The imaginary modulus data agreed well with the KWW formulism, and the resulting stretching coefficient was less than unity, signifying the occurrence of a non-Debye-type relaxation process. The Nyquist plot comprising depressed semicircles was modelled by an equivalent circuit model with the configuration (R_G_Q_G_) (R_GB_Q_GB_), revealing the bulk and interfacial contributions of the microconstituents to the dielectric relaxation and electrical conduction mechanisms in the sample. The frequency exponent calculated from the fit of Jonsher's power law to the AC conductivity spectrum suggested that an overlapping large polaron hopping mechanism was responsible for conduction in Zn_0.5_Cd_0.5_Fe_2_O_4_ spinel ferrite. The real and imaginary parts of the dielectric constant were found to vary directly with temperature, reaching maximum values of approximately 125 and $22\times 10^{3}\text{,}$ respectively. The Tan loss also exhibited an increasing trend with temperature, reaching a maximum value of approximately $15\times 10^{4}$ at 438 K. The tan loss and dielectric constant spectra confirmed the existence of a thermally activated hopping mechanism in the sample.

## Data availability

Data will be made available on request.

## CRediT authorship contribution statement

**Raheel Mumtaz:** Validation. **Waqar Hussain Shah:** Writing – original draft, Data curation, Conceptualization. **Yousaf Iqbal:** Writing – review & editing, Supervision, Project administration. **Hayat Ullah:** Visualization, Formal analysis. **Ghulam Asghar:** Formal analysis. **Mubushar Hussain:** Validation, Investigation. **Mostafa R. Abukhadra:** Formal analysis. **Ahmed M. El-Sherbeeny:** Resources, Funding acquisition.

## Declaration of competing interest

The authors declare that they have no known competing financial interests or personal relationships that could have appeared to influence the work reported in this paper.
